# Next generation oncolytic viruses expressing PADI1 and TIMP2 exhibit anti-tumor activity against melanoma in nude and humanized mouse models

**DOI:** 10.1016/j.omto.2023.01.002

**Published:** 2023-01-13

**Authors:** Lukasz Kuryk, Anne-Sophie W. Møller

**Affiliations:** 1 Targovax ASA, Clinical Science, Vollsveien 19, NO-1366 Lysaker Oslo, Norway; 2 National Institute of Public Health NIH – National Research Institute, Department of Virology, Chocimska 24, 00-791 Warsaw, Poland

**Keywords:** melanoma, arginine deprivation, oncolytic adenovirus, TIMP2, humanized NOG tumor model, arginine deiminase, PADI1, animal studies, immunotherapy, cancer models

## Abstract

Immunotherapy of metastatic melanoma (MM) has vastly improved the longevity of only a minority of patients. To broaden the repertoire of agents against MM, we investigated the effectiveness of locally interrupting tumor blood endothelial cell proliferation and angiogenesis, arginine deprivation, or both on the growth of melanoma by constructing and characterizing the effectiveness of four oncolytic adenoviruses. ONCOS-207 (which expressed tissue inhibitor of metalloprotease type 2 [TIMP2]), ONCOS-209 (which expressed peptidyl arginine deiminase [PADI1]), and ONCOS-210 and ONCOS-212 (which expressed both TIMP2 and PADI1) exhibited oncolytic activity against four melanoma cell lines *in vitro*. ONCOS-212 treatments significantly inhibited tumor growth in an A2058 tumor model in nude mice compared with vehicle control. The inhibitory effects of the two transgenes of ONCOS-212 on tumor growth appeared to be synergistic. These viruses also significantly inhibited tumor growth in a humanized NOG model of melanoma (A2058 xenograft). All viruses significantly increased the percentage of activated CD8+ T cells in the tumor-infiltrating lymphocytes. The abscopal effect of ONCOS-212 treatments in the A2058 tumor challenge model in hNOG mice supports the hypothesis that the human immune response contributes to the anti-tumor activity of ONCOS-212. These results support the further development of ONCOS-212 for cancer treatment.

## Graphical abstract

**Figure:**
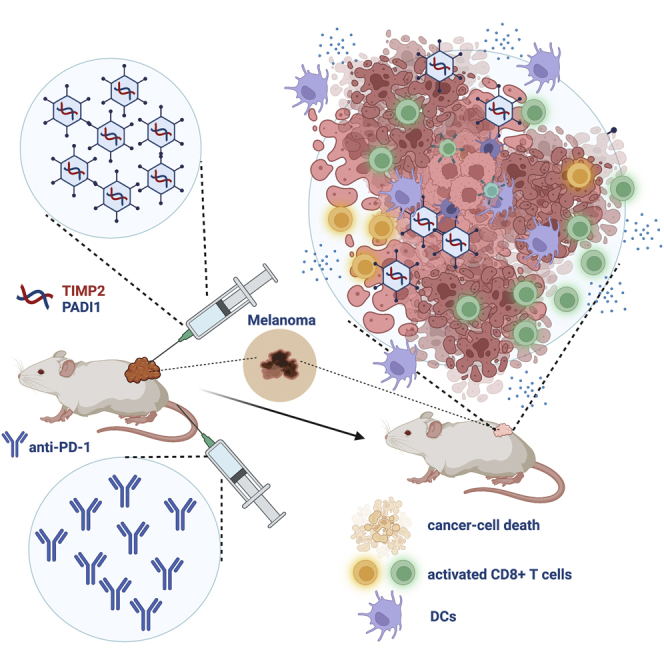

## Teaser

Dual-expressing (TIMP2 + PADI1) oncolytic adenoviruses had significantly higher anti-tumor activity than single-transgene oncolytic viruses and vehicle control. Treatment with the viruses had significantly increased the percentage of activated cytotoxic T cells and reactivation of T cells with anti-tumor activity during treatment of melanomas with immune checkpoint inhibitors.

## Introduction

Melanoma is the fifth most common cancer diagnosed in the United States, totaling approximately 100,350 cases annually.^1^ The 5-year survival rate of people diagnosed with melanoma ranges from 98% for localized melanoma to 23% for metastatic melanoma (MM).^1^ Exposition of the skin with solar UV radiation is the main cause of skin cancer development.^2^^,^^3^ Although immune checkpoint inhibitors (CPIs) have greatly increased the survival of some patients with MM by several years, many patients show resistance to CPIs during the initial treatments.^4^ Furthermore, about 25% of the initial set of responders, despite ongoing CPI treatment, relapse with acquired resistance to CPI treatments.^5^^,^^6^ Thus, the development of novel strategies for targeting MM is warranted.^7^ To broaden the repertoire of mechanisms of action of agents against melanoma, we investigate the effectiveness of locally interrupting tumor blood endothelial cell proliferation and angiogenesis, arginine deprivation, or both on the growth of melanomas.

A regulator of the extracellular matrix (ECM), tissue inhibitor of metalloprotease type 2 (TIMP2), is overexpressed in regressing parts of melanomas.^8^ TIMP2 is one of four TIMPs that regulate the extracellular matrix in the tumor environment by modulating the catalytic activity of a family of zinc-dependent endoproteases called the matrix metalloproteases (MMPs). The degradation of the ECM by MMPs can stimulate tumor growth and an influx of blood vessel growth (angiogenesis), release sequestered growth factors, and allow migration of metastatic tumor cells/clumps.^9^ TIMPs not only inhibit the activity of various MMPs but also can modulate angiogenesis, proliferation, and apoptosis by a MMP-independent mechanisms. TIMP2 can bind the integrin α3β1 on endothelial cells and can inhibit endothelial cell proliferation, migration, and angiogenesis in an MMP-independent manner.^9^ Furthermore, TIMP2 can inactivate VEGFR2 and FGFR1 on endothelial cells, which reduces their proliferation and angiogenesis. Its interaction with VEGFA increases cell-to-cell contact and decreases vascular permeability. Local overexpression of TIMP2 in tumor tissue may provide anti-tumor activity in melanomas.

Arginine deprivation can induce autophagy, apoptosis, antiangiogenic effects, and reduced production of nitric oxide (NO) in arginine-dependent cancers, including melanoma,^10^^,^^11^ mesothelioma,^12^ colon cancer,^13^ and prostate cancer.^14^ Approximately 70% of primary melanomas do not express argininosuccinate synthetase-1 (ASS1), which produces arginine.^10^ The prognosis of patients with malignant melanoma treated with pegylated arginine deiminase (ADI-Peg) correlated with the baseline status of ASS1 expression: patients who had ASS1-negative MM and received four doses of ADI-Peg of 320 IU/m^2^ had a significantly longer survival (26.5 months) than patients with ASS1-positive MM (8.5 months).^10^ Feun et al.^10^ concluded that both the baseline ASS1-negative MM phenotype and the dose of ADI-Peg20 contributed to the 70% clinical benefit rate. Local expression of peptidyl arginine deiminase type I (PADI1) in tumors can provide enhanced tumor apoptosis, antiangiogenic effects, and reduced production of nitric oxide induced by arginine deprivation.

Targeted delivery of these proteins with oncolytic adenoviruses with potential anti-tumor activity to the melanomas may reduce any potential undesired off-target effects. Oncolytic viruses are engineered to replicate in cancer cells but not healthy cells.^15^^,^^16^^,^^17^^,^^18^^,^^19^^,^^20^^,^^21^^,^^22^ Oncolytic adenoviruses provide an anti-tumor platform that not only can lyse tumor cells but also can deliver exogenous genes that augment anti-tumor activity using distinct mechanisms of action.^23^^,^^24^^,^^25^^,^^26^^,^^27^^,^^28^^,^^29^ Several oncolytic adenoviruses that express granulocyte-macrophage colony stimulating factor (GM-CSF)^30^ or OX40L^31^ have shown promise in treatment of melanoma in animal models and phase I clinical trials.^32^^,^^33^^,^^34^^,^^35^^,^^36^^,^^37^^,^^38^ The oncolytic adenoviral backbone includes two modifications that augment its efficacy and selectivity for melanoma tumor cells. First, its chimeric fiber knob (Ad5 with binding loop of Ad3 [Ad5/3]) binds to the membrane protein desmoglein,^39^ which is frequently overexpressed on melanoma cells.^40^^,^^41^ Second, the E1A gene contains the 24 bp deletion that limits its replication to tumor cells containing a defective retinoblastoma (Rb) pathway^40^ found in many melanomas. Here we compare the efficacy of oncolytic adenoviruses that express peptidyl arginine deiminase type I, TIMP2, or both. After assessing their *in vitro* activity, we compare their *in vivo* activity not only in a xenogeneic model of melanoma in nude mice but also in the human hematopoietic stem cell engrafted NOG mice (hu-NOG mice)^33^^,^^34^^,^^42^ to more closely mimic the induction of human anti-tumor immune responses. Because an improved prognosis of patients with MM is associated with higher numbers of tumor-infiltrating lymphocytes (TILs), especially CD8+ T cells, we also compared the profiles of the different cell types (effector T lymphocytes, regulatory T lymphocytes [Treg], macrophages, dendritic cells, and natural killer cells) in the TILs in the different treated groups.

## Results

Four oncolytic adenoviruses with the Δ24 deletion in the E1A gene and the chimeric Ad5/3 fiber knob were engineered to express TIMP2 (ONCOS-207), PADI1 (ONCOS-209), or both PADI1 and TIMP2 (ONCOS-210 and ONCOS-212) in the E3 region (Table S1). Both transgenes are intracellular proteins without signal protein sequence and thus are released to the cytosol and tumor microenvironment. ONCOS-210 had the expression cassette PADI1-P2A-TIMP-2, whereas ONCOS-212 used the CMV promoter to provide constitutive expression of the two transgenes throughout infection: CMV promoter-PADI1-IRES-TIMP-2-SV40 poly A signal (Table S1). As expected, ONCOS-207-, ONCOS-210-, and ONCOS-212-infected cells expressed TIMP-2, and ONCOS-209, ONCOS-210, ONCOS-212 expressed PADI1 (Figure S1).

**Figure 1:**
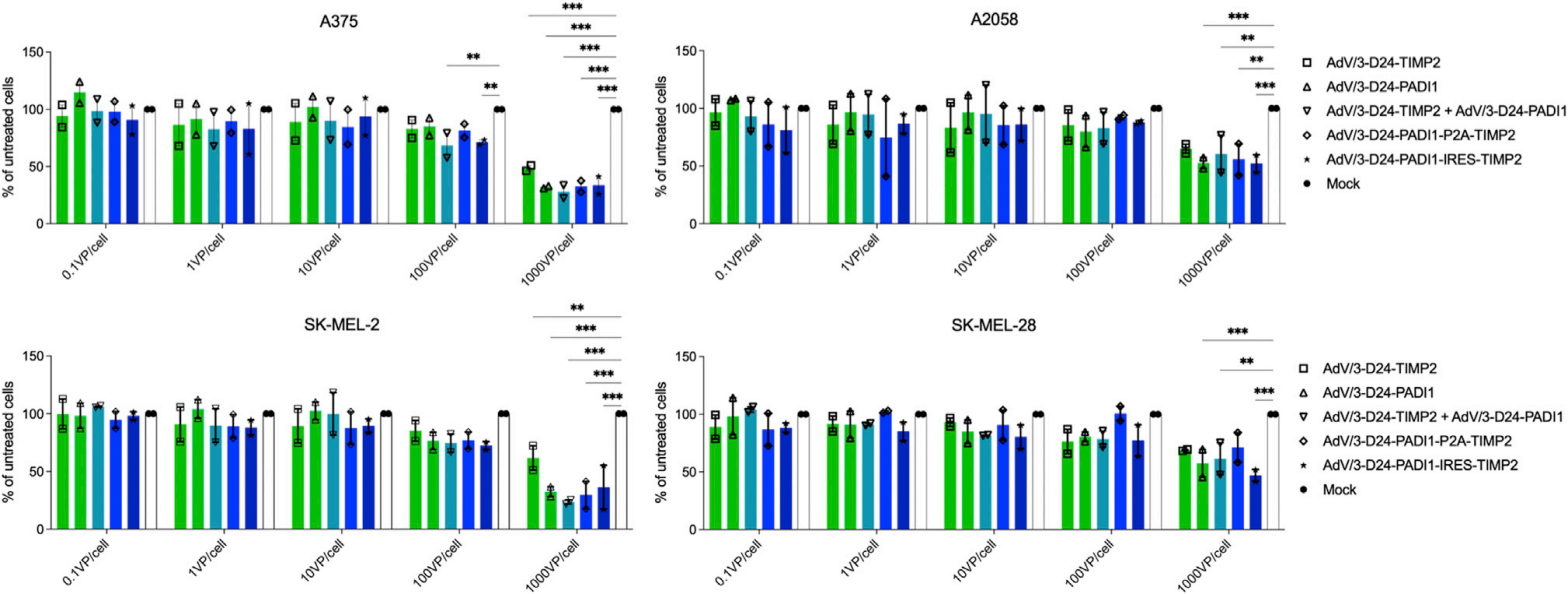
Viability of the four melanoma cells lines at 72 h after vehicle (media) or indicated oncolytic adenovirus treatments Viability of A375 and A2058, SK-Mel-2, and SK-Mel-28. Results are expressed as mean ± SEM. Analyses were run in duplicates. Two-way ANOVA with Tukey’s multiple comparison’s test was used. ∗∗p ≤ 0.01 and ∗∗∗p ≤ 0.001.

The oncolytic activity of the four oncolytic adenoviruses and a combination therapy (ONCOS-207 + ONCOS-209) viral preparations were assessed against four human melanoma cell lines (A2058, A375, SK-MEL-2, and SK-MEL-28) that are negative for expression of argininosuccinate synthetase-1.^43^^,^^44^ Treatment with ONCOS-207, ONCOS-209, the combined ONCOS-207 + ONCOS-209, ONCOS-210, or ONCOS-212 at 1,000 viral particles (VP)/cell significantly reduced cell viability at 72 h in the four melanoma cell lines compared with vehicle (media) treatment (Figure 1). The most sensitive melanoma cell line to these oncolytic viruses was A375, followed by SK-MEL-2, A2058, and SK-MEL-28 cell lines.

**Figure 2:**
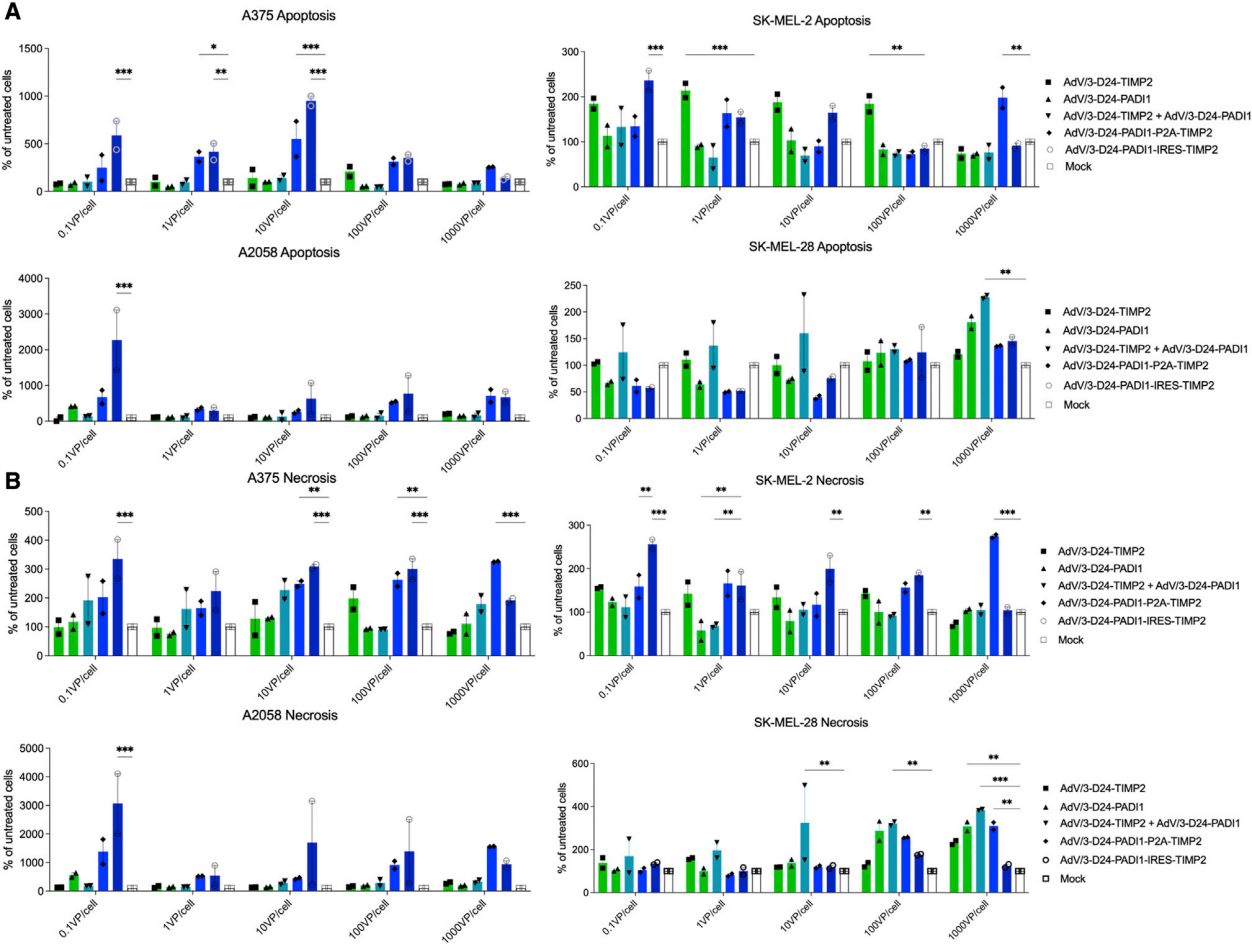
Apoptosis and necrosis induced by the oncolytic viruses (A and B) Relative percentage of cells undergoing apoptosis (A) and necrosis (B) at 72 h after induction by viral treatment in the indicated melanoma cell lines. Results are expressed as mean ± SEM. Analyses were run in duplicates. Two-way ANOVA with Tukey’s multiple-comparisons test was used. ∗p ≤ 0.05, ∗∗p ≤ 0.01, and ∗∗∗p ≤ 0.001.

Induction of apoptosis and necrosis by four oncolytic viruses were investigated. As expected, vehicle (media) treatment exhibited a similar percentage of apoptosis and necrosis as the untreated cells. Regarding apoptosis, ONCOS-212-treated A2058, A375, and SK-MEL-2 cells (0.1, 1, and 10 VP) showed significantly more apoptosis than the vehicle-treated cells (Figure 2). ONCOS-212 also induced significantly more necrotic cell death in A2058, A375, and SK-MEL-2 cells (0.1 and 10 VP) than the vehicle treatment (Figure 2). Note that the relative percentage increase of apoptosis and necrosis for oncolytic adenovirus-induced cell death was higher in A2058 (ranging up to a mean of 3,000% of untreated cells) than those observed in the A375, SK-MEL-2, and SK-MEL-28 cell lines (Figure 2).

**Figure 3:**
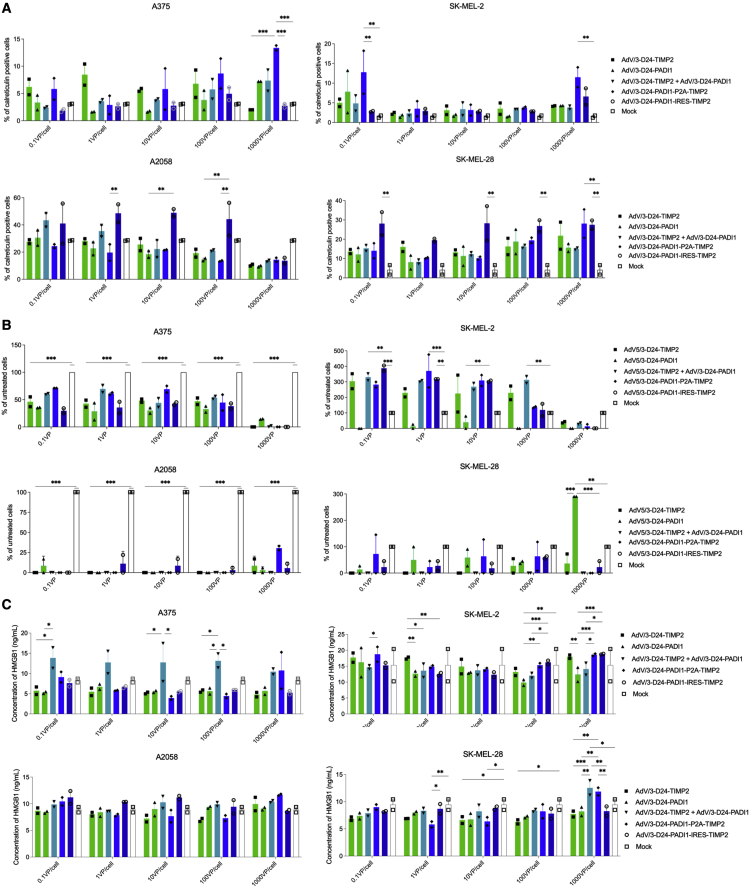
Immunogenic death induced in melanoma cell lines at 72 h after viral treatment (A) Calreticulin exposure. Relative percentage of cells positive for calreticulin exposure at 72 h after viral treatment compared with vehicle (media) treatment in the indicated melanoma cell lines. Calreticulin exposure was detected by staining with anti-calreticulin antibodies and assessed using flow cytometry. (B) ATP release. Relative percentage of ATP released at 72 h after induction by viral treatment compared with mock treatment in the indicated melanoma cell lines was measured by using the ATP assay kit. (C) HMGB1 release. HMGB1 concentration released at 72 h after induction by viral treatment compared with mock treatment in the indicated melanoma cell lines was measured using the ELISA kit. Results are expressed as mean ± SEM. Analyses were run in duplicates. Two-way ANOVA with Tukey’s multiple-comparisons test was used. ∗p < 0.05, ∗∗p ≤ 0.01, and ∗∗∗p ≤ 0.001.

Immunogenic cell death after oncolytic viral treatment was assessed by calreticulin exposure, ATP secretion, and HMGB1 release. The effect of viral treatments on calreticulin exposure was dependent on the virus, dose, and cell line (Figure 3). ONCOS-212 treatment (10, 100, and 1,000 VP/cell) and ONCOS-210 (1,000 VP/cell) significantly increased calreticulin exposure in SK-MEL-28 compared with vehicle treatment (p < 0.01). Compared with vehicle treatment, the four viruses affected ATP secretion in the treated A375 and A2058 cells but not SK-MEL-2 and SK-MEL-28 cells (Figure 3). Unexpectedly, the viral treatments at all doses significantly inhibited ATP secretion in A375 and A2058 cells. The quantity of ATP secretion from the four vehicle-treated cell lines was the highest in SK-MEL-2 cells. Compared with vehicle treatment, no viral treatment significantly altered HMGB1 secretion (Figure 3).

### Comparative efficacy in A2058 tumor model in nude BALB/c mice

**Figure 4:**
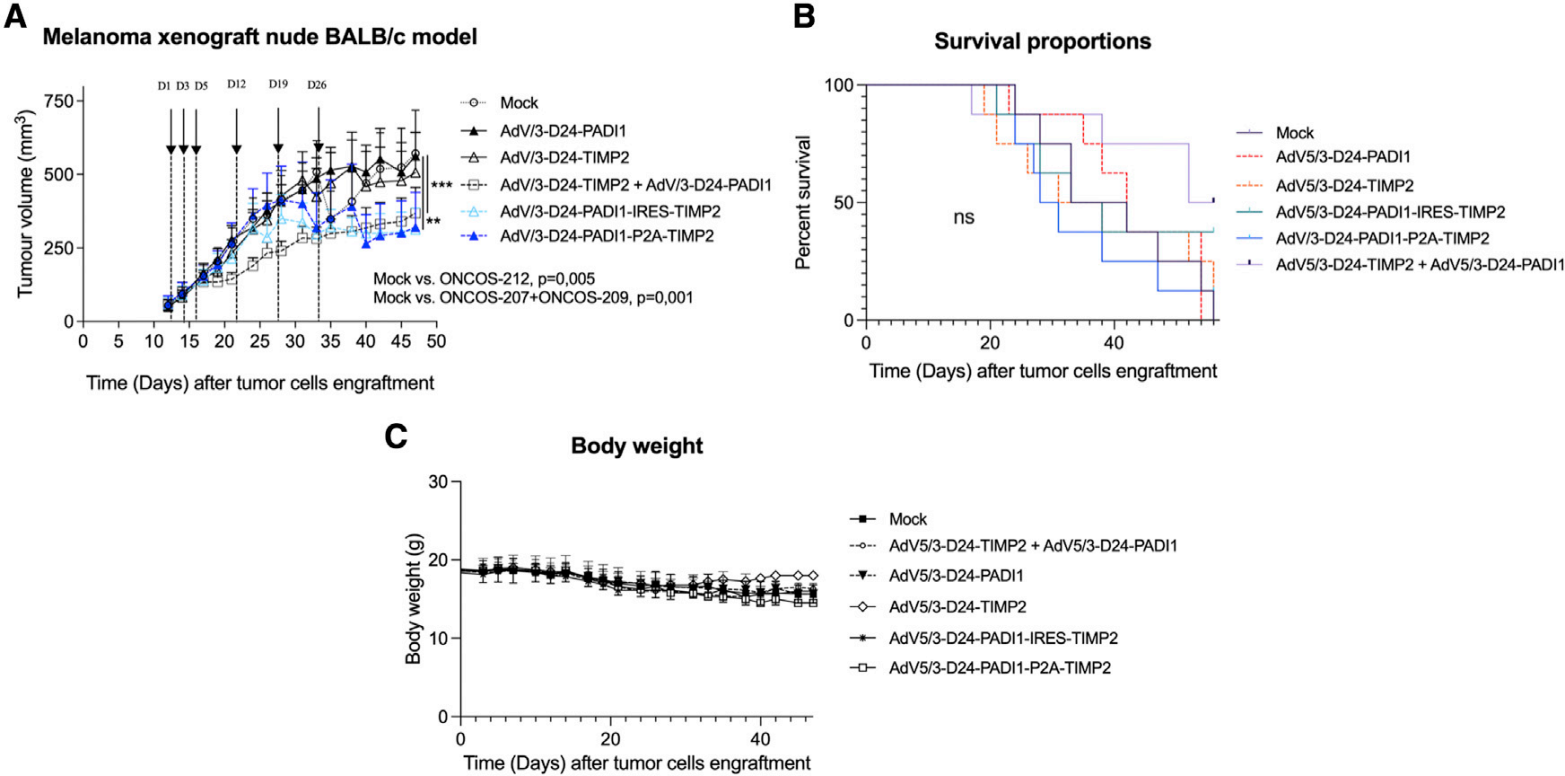
Efficacy of oncolytic adenoviral treatments in an A2058 tumor model in the nude BALB/c model Six treatments (PBS or 2.5 × 10^6^ VP/tumor treatment, both tumors were treated) were administered intratumorally on days 1, 3, 5, 12, 19, and 26 after randomization. The tumor volumes and clinical health scores were monitored three times per week. (A) The effect of vehicle or adenoviral treatments on tumor growth. Results are expressed as mean ± SEM. One-way ANOVA with Tukey’s multiple-comparisons test was used, with n = 6–16 tumors per group. ∗∗p ≤ 0.01 and ∗∗∗p ≤ 0.001. (B) Kaplan-Meier survival curve of oncolytic adenoviral-treated A2058 melanomas in the nude BALB/c model. ns, non-significant. (C) Body weight of the vehicle and treated nude BALB/c mice. Results are expressed as mean ± SEM.

The efficacy of the four viruses and the combination therapy (ONCOS-207 + ONCOS-209) were compared with vehicle (PBS) treatment in an A2058 tumor model in nude BALB/c mice. Mean tumor volumes in the left and right flanks ranged from 101 to 135 mm^3^ and from 64 to 81 mm^3^ at randomization, respectively. Body weight slightly decreased after tumor cell engraftment in all groups (Figure 4C).

The mean tumor volumes of the ONCOS-210 and ONCOS-212 group and the combination ONCOS-207 + ONCOS-209 group were significantly smaller than those of the vehicle-treated, ONCOS-207, and the ONCOS-209 groups (p < 0.01; Figure 4A). Because nude mice have defective adaptive immune responses, the observed anti-tumor effects have not included any contribution from the hosts’ adaptive immune responses.

### Synergy assessment of tumor volume inhibition

The reduced tumor volumes observed in the groups treated with ONCOS-210, ONCOS-212, and the combination (ONCOS-207 + ONCOS-209) groups were greater than expected as determined by the fractional tumor volume (FTV) method (Table S2). The calculated synergism was observed on day 21 and day 33 post-treatment initiation.

Kaplan-Meier survival curves are depicted in Figure 4B. The median survival times of vehicle treatment (37.5 days), ONCOS-212 (35.5 days), ONCOS-207 (34.5 days), and ONCOS-209 (42 days) were similar. The combination therapy (ONCOS-207 + ONCOS-209) group had longer median survival (54 days) than the vehicle control group.

### Efficacy in A2058 tumor model in humanized NOG mice

The A2058 melanoma model in the humanized NOG mice provides an *in vivo* model that more closely mimics human immune responses^45^ expected during administration in the clinic. After humanization of the NOG mice, followed by A2058 implantation and randomization on the basis of humanization rate and tumor volumes (Figure S2), three hypotheses were tested using the indicated dosing schedule in Figure S3: (1) the oncolytic viruses expressing the two transgenes would be more effective than an oncolytic expressing one transgene, (2) ONCOS-212 efficacy is increased with a 200-fold higher dose, and (3) ONCOS-212 treatment of A2058 can reduce growth of subsequent challenge of A2058 cells as an assessment of induction of anti-tumor immune response.

**Figure 5:**
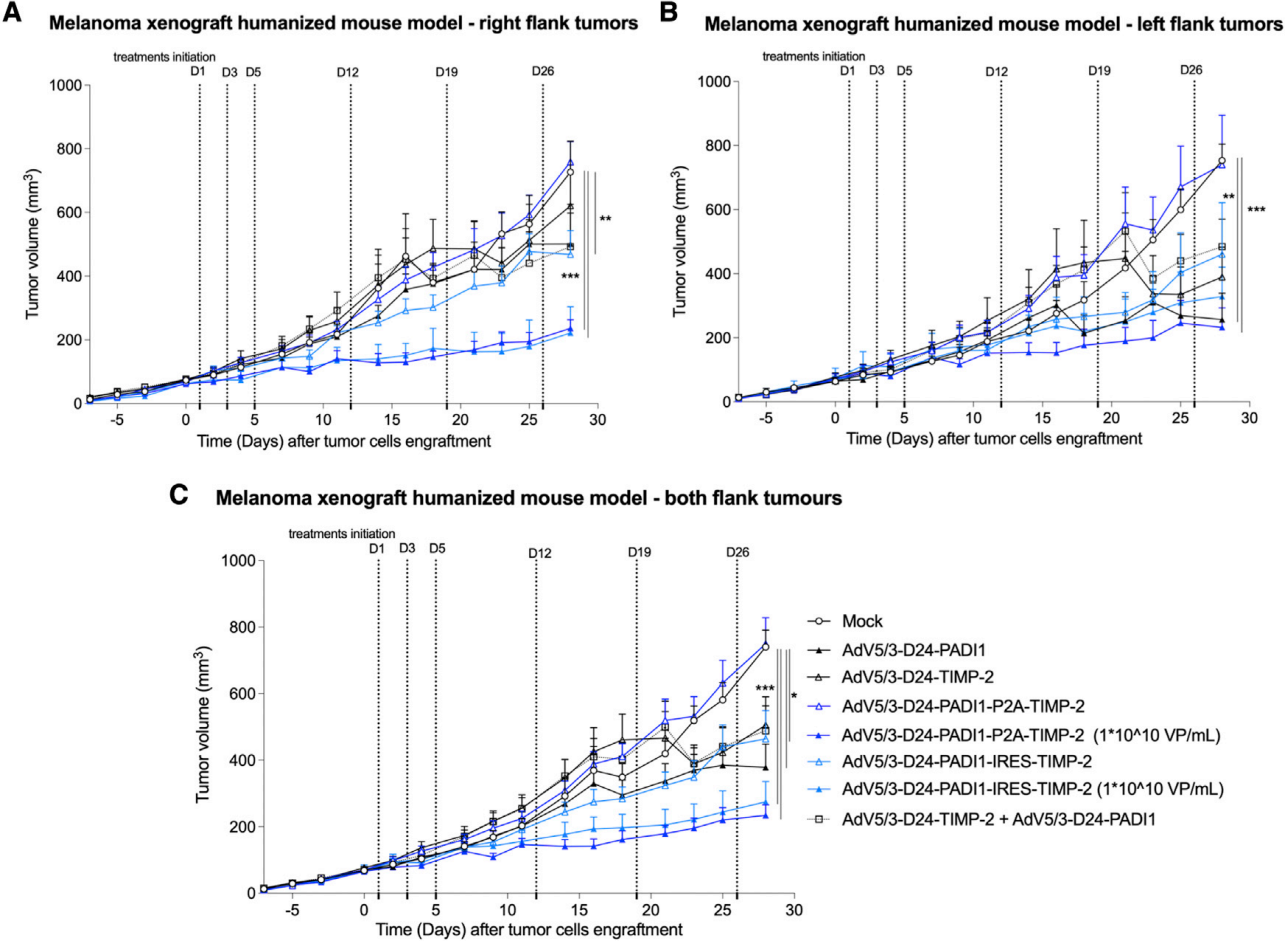
Effect of oncolytic viruses on A2058 tumor growth in hNOG model Fourteen days after tumor inoculation and one day after randomization into groups, treatments (PBS or 2.5 × 10^6^ VP/tumor treatment) were administered intratumorally (both tumors were treated) on days 1, 3, 5, 12, 19, 26 (Figure S3). The tumor volumes and clinical health scores were monitored three times per week. (A–C) The average tumor volumes of the (A) right flank tumors, (B) left flank tumors, and (C) combined right and left flank tumors are displayed on the indicated days. Results are expressed as mean ± SEM. One-way ANOVA with Tukey’s multiple-comparisons test was used, with n = 3–8 mice per group or 6–16 tumors per group. ∗p < 0.05, ∗∗p ≤ 0.01, and ∗∗∗p ≤ 0.001.

The groups treated with 2.5 × 10^6^ VP/tumor treatment of ONCOS-212, ONCOS-207, ONCOS-209, and the combination ONCOS-207 + ONCOS-209 (but not ONCOS-210) had significantly reduced tumor volumes on day 28 (end of study) compared with the vehicle-treated group in the A2058 tumor model in humanized NOG mice (Figure 5). As example, compared with vehicle control, ONCOS-212 had significantly reduced tumor volume in the right tumor group (p ≤ 0.01). The FTV method did not detect synergistic activity in the reduced tumor volume in the groups treated at 2.5 × 10^6^ VP/tumor with ONCOS-212, ONCOS-210, or the combination ONCOS-207 + ONCOS-209 (data not shown).

Furthermore, high-dose ONCOS-212 (5 × 10^8^ VP [1 × 10^10^ VP/mL]/tumor treatment) significantly reduced the tumor growth in right flank tumors, left flank tumors, and both flank tumor groups compared with the vehicle control group (p ≤ 0.001, p ≤ 0.001, and p ≤ 0.05, respectively) (Figure 5). Because the effects of the single viruses were investigated only at the lower dose (2.5 × 10^6^ VP/tumor dose), the FTV method for detecting synergism was not applied to the high-dose ONCOS-212 and ONCOS-210 groups. These data further support the development of ONCOS-212 against human melanoma cells.

**Figure 6:**
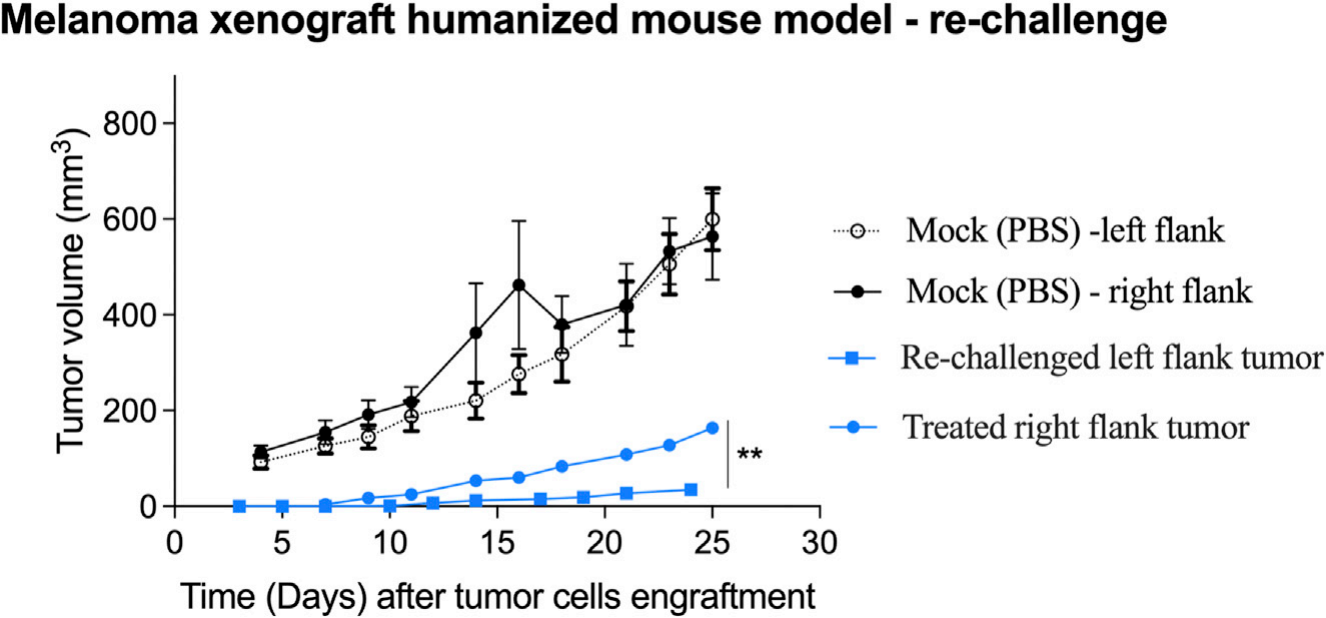
ONCOS-212 induced abscopal effect in re-challenge study in A2058 tumor model in humanized NOG model Intratumoral ONCOS-212 treatment began 14 days after A2058 cells (5 × 10^5^) were implanted in the right flank tumors (considered day 1). ONCOS-212 treatment was also injected intratumorally on days 1, 3, 5, 12, 19, and 26, as illustrated in Figure S3B. On day 18, A2058 cells (5 × 10^5^) were implanted in the left flank. Tumor volumes (square millimeters) and clinical health scores were assessed three times per week. Results are expressed as mean ± SEM. The t test was used. ∗∗p ≤ 0.01.

Metastases remain a major challenge in treatment of late-stage melanoma. Induction of an immune response in the treated tumor nodule may reduce growth in a distant nodule and is often called an abscopal effect. To stimulate and detect a potential ONCOS-212-induced immune response to A2058 tumor cells *in vivo*, we assessed whether ONCOS-212 treatment of A2058 tumors could alter the growth rate of a subsequent A2058 challenge. The A2058 inoculums were smaller (5 × 10^5^ cells) to allow the longer duration of study, and the challenge was administered 18 days later to provide sufficient time to develop an adaptive immune response. As expected, the A2058 tumor in this study grew slower and reached a mean tumor volume of only 160 mm^3^ day 28 after A2058 engraftment because of the smaller inoculum. The A2058 tumor challenge showed significantly slower growth (p ≤ 0.01) during the 24 day duration of the study than the treated right (Figure 6). The significant abscopal effect of ONCOS-212 in the A2058 tumor challenge model in hNOG mice supports the hypothesis that the human immune response contributes to the anti-tumor activity of ONCOS-212.

### Oncolytic viral treatment increased PD-L1 and PD-L2 expression on A2058 tumors

All oncolytic viral treated groups had significantly fewer A2058 tumor cells per tumor tissue than the vehicle control group (data not shown).

Expression of program cell death ligand 1 (PD-L1) and PD-L2 on primary melanoma cells are being investigated as a biomarker for sensitivity to anti-PD-1-based immunotherapies. Some cancer treatments including the oncolytic adenovirus ONCOS-102 can augment PD-L1 expression on melanoma cells.^33^ Thus, the effects of these oncolytic viruses on the expression of PD-L1 and PD-L2 on A2058 tumor cells were investigated by using flow cytometry. ONCOS-209, ONCOS-207 + ONCOS-209, and ONCOS-212 groups but not the ONCOS-210 groups significantly increased the percentage of PD-L1-expressing A2058 tumor cells (Figure S4A). Furthermore, the ONCOS-207, ONCOS-209, ONCOS-207 + ONCOS-209, ONCOS-210, and ONCOS-212 groups significantly increased the percentage of PD-L2 + A2058 tumor cells (Figure S4B). These data raise the prospect of investigating combination therapy with anti-PD-1-based immunotherapies.

### Tumor-infiltrating lymphocytes

TILs can vary in distribution and density in the tumor, relative mix of the distinct cell types (e.g., CD4+ T cells, CD8+ T cells, Treg; proinflammatory macrophages vs regulatory macrophages) and activation states. Evidence is mounting that the TIL profile can act as an independent predictive factor of a patient’s prognosis. As the prognosis of patients with MM is improved with higher CD8+ T cells in the TILs,^46^ the numbers of human CD45+ cells, CD3+ T cells, CD4+ T cells, CD8+ T cells, and T regulatory cells per gram of tumor tissue in each treated group was analyzed via flow cytometry. Only ONCOS-210-treated (2.5 × 10^6^/tumor) A2058 from hNOG mice showed significantly higher numbers of CD8+ T cells per gram of tumor tissue than the vehicle control group (Figure S5). The higher numbers of TILs observed in ONCOS-210-treated (2.5 × 10^6^/tumor) A2058 tumors may have contributed to the unexpected large tumor volumes, which were not reduced compared with vehicle-treated tumors. Interestingly, PD-1 expression was significantly higher in CD8+ T cells, but not CD4+ T cells in the TILs from the ONCOS-210 compared with the vehicle-treated group (Figure S6). These data imply that the efficacy of these viruses combined with anti-PD-1 immunotherapy warrants investigation.

**Figure 7:**
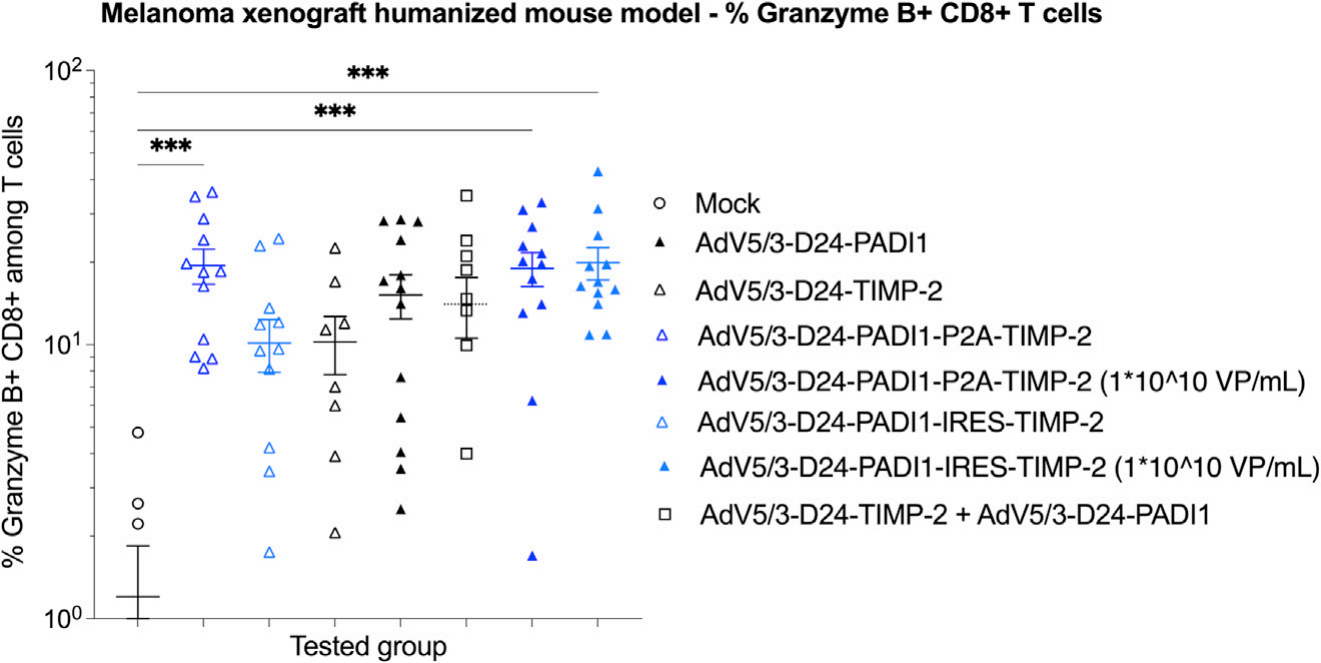
Granzyme B expression in infiltrated CD8+ T cells Data from right and left flank tumors in a group were combined. n = 6–12 tumors per group. Individual data and mean ± SEM are presented for each group. One-way ANOVA with Tukey’s multiple-comparisons test was used. ∗∗∗p ≤ 0.001.

The percentage of detectable granzyme B+ CD8+ T cells is often considered to correlate with the number of activated CD8+ T cells (commonly referred to as cytotoxic T cells).^46^ The ONCOS-210 and ONCOS-212 treatment groups expressed significantly higher percentages of granzyme B+ CD8+ T cells in the TILs of their respective day 28 A2058 tumors than vehicle control (Figure 7), indicating that immune mechanisms involving activated CD8+ T cells contributed to the reduction in tumor volumes.

**Figure 8:**
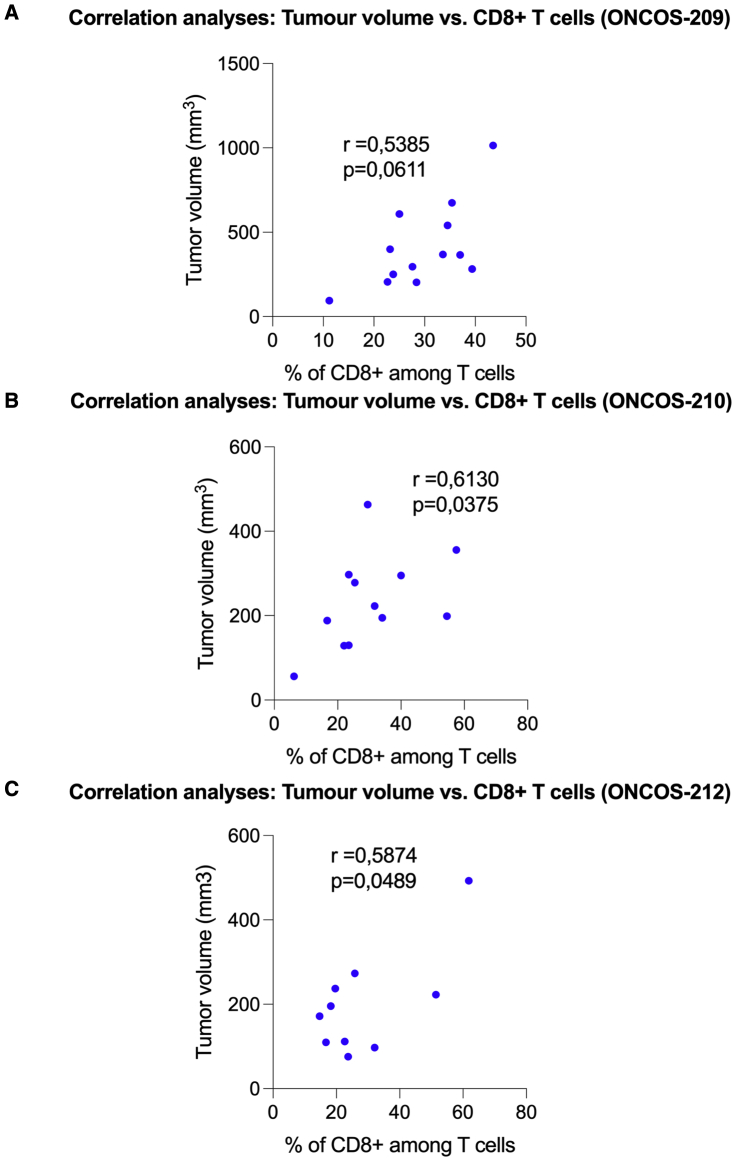
Spearman correlation coefficient was used to identify potential relationships between individual tumor volume and percentage of CD8+ T cells in the tumor-infiltrating lymphocytes per gram of tumor tissue (A–C) Granzyme B expression in infiltrated CD8+ T cells. Data from right and left flank tumors in a group were combined. N = 6-12 tumors per group. Individual data and mean ± SEM are presented for each group. One-way ANOVA with Tukey’s multiple comparison’s test was used. ∗∗∗ P ≤ 0.001.

The repeated administration of tested oncolytic viruses showed a moderate relationship between tumor volume and percentage of CD8+ T cells in the tumor-infiltrating T cells, with r values ranging between 0.54 and 0.61 (Figure 8).

## Discussion

Oncolytic adenoviruses with the E1A Δ24 deletion provide an anti-tumor platform that specifically replicates in tumor cells with impaired retinoblastoma pathways, including most melanomas. Although systemic administration of oncolytic adenoviruses in humans can reach metastases, García et al.^47^ reported that their oncolytic adenoviruses had not induced tumor regression and indicated the importance of arming the oncolytic adenovirus to express exogenous proteins that provide and elicit anti-tumor activity. Both arginine deprivation which can be induced by PADI1 and TIMP2 which modulates MMP activity and thus ECM integrity are associated with regression.^48^ Arginine deprivation exhibits anti-tumor activity against melanoma.^10^ In this study, our four novel oncolytic adenoviruses armed to express one or both PADI1 and TIMP2 exhibited varied levels of cytotoxicity against four melanoma cell lines *in vitro* in a dose-dependent manner. The ONCOS-210 and ONCOS-212 oncolytic adenoviruses, which express both PADI1 and TIMP2, and the combination (ONCOS-207 + ONCOS-209) significantly inhibited A2058 tumor growth in the nude mouse model compared with the vehicle-treated group and the single-transgene vectors ONCOS-207 and ONCOS-209. The efficacy of ONCOS-210, ONCOS-212, and the combination ONCOS-207 + ONCOS-209 in the A2058 tumor model in nude mice indicated a synergistic anti-tumor effect over the groups treated with the single-transgene oncolytic adenoviruses ONCOS-207 or ONCOS-209, indicating the superiority of dual-armed oncolytic adenoviruses.

Oncolytic viral treatment of the A2058 tumors in humanized NOG model induces immune responses that probably more closely mimics human immune responses in the clinic than those in syngeneic mouse models.^45^ The treatment groups with ONCOS-207, ONCOS-209, ONCOS-212, the combination ONCOS-207 + ONCOS-209, but not ONCOS-210 (2.5 × 10^6^ VP/tumor treatment) had significantly reduced tumor volumes on day 28 (end of study) compared with the vehicle-treated group. Furthermore, high-dose ONCOS-212 and ONCOS-210 (5 × 10^8^ VP/tumor treatment) reduced the tumor growth compared not only with the vehicle control group but also to both treatment groups that received 2.5 × 10^6^ VP/tumor of ONCOS-210 and ONCOS-212, indicating that the higher dose was associated with greater efficacy. The reduced anti-cancer effect (ability to control the tumor growth) observed in mice treated with ONCOS-210 (2.5 × 10^6^ VP/tumor treatment) might be related to lower expression of encoded transgene such as PADI1 (Figure S1), which presumably relates to low virus dose used in the study (2.5 × 10^6^ VP/tumor). Additionally, we can speculate that the expression cassette under the exogenous promoter: CMV in ONCOS-212 enhanced expression of PADI1 in comparison with the vector ONCOS-210, resulting in higher expression of the transgene.

The prognosis of melanoma patients is associated with the numbers, types, activation states and distribution of the TILs.^46^^,^^49^ Metastatic melanomas have evolved with accompanying lymphocytes that often suppress or derail an anti-tumor immune response via multiple mechanisms.^46^ As a major advantage for anti-tumor therapy, oncolytic adenovirus treatments usually trigger immunogenic cell death as they lyse the tumor cells and release their progeny.^15^^,^^50^ ONCOS-212 treatment induced significantly more apoptosis and necrotic cell death in A2058 cells than vehicle *in vitro*. Arginase treatment of acute lymphoblastic leukemia also induced necrosis and apoptosis.^51^^,^^52^ Whereas treatment with these viruses did not significantly induce a greater expression of HMGB1 or increase ATP release, treatments did significantly activate a higher percentage of TILs, as discussed below.

The prognosis of patients with MM is improved with greater numbers of activated CD8+ T cells in the TILs.^46^ The percentage of detectable granzyme B+ CD8+ T cells is often considered to correlate with the number of activated CD8+ T cells. Although most of these oncolytic adenoviruses had not significantly increased the numbers of human CD8+ T cells per gram of tumor tissue, the tumors of all viral treatment groups exhibited significantly higher percentages of granzyme B+ CD8+ T cells in the TILs of their respective day 28 A2058 tumors than vehicle control, indicating that immune mechanisms involving activated CD8+ T cells contributed to the significant reduction in tumor volumes. Treatment with these viruses had significantly increased the percentage of activated cytotoxic T cells, which often correlates with improved outcomes in patients with melanoma clinical trials. The repeated administration of high-dose ONCOS-212 showed a moderate correlation between tumor volume and percentage of CD8+ T cells in the tumor-infiltrating T cells (r = 0.5874).

TIMP2 or arginine deprivation localized to the melanoma tumor cell environment by the treatment with TIMP2- or PADI1-expressing oncolytic adenoviruses (ONCOS-209, ONCOS-210, and ONCOS-212) exhibited similar reduction of weight gain as the vehicle-treated group, suggesting the possibility of minimal systemic toxicity.

Integrating oncolytic Ad into combination therapy may augment the efficacy of cancer treatments and may broaden the number of responding patients.^53^^,^^54^ As PD-1 expression was significantly increased in the CD8+ T cells of both doses of ONCOS-212-treated tumor groups compared with the vehicle-treated group, the possibility that combination therapy with anti-PD-1 immunotherapeutic agents and ONCOS-212 may extend the benefits of anti-PD-1 therapy to a broader base of patients with melanoma warrants investigation. For example, recently a patient who had MM, had failed immunotherapy, and was treated with pegylated recombinant arginase that induced arginine deprivation experienced a complete remission lasting ≥30 months.^55^

### Limitations

As these four melanoma cells did not express ASS1 they might be more sensitive to arginine deprivation induced by local expression of PADI1. Some primary melanomas express ASS1 and appear less sensitive to arginine deprivation as evidenced in a clinical trial of ADI-Peg.^10^ Comparison of the efficacy of ONCOS-212 in subgroups of patients with melanomas that differ in ASS1 status probably could inform the susceptible cohorts. Another limitation includes the probable HLA mismatches between the A2058 tumor cells and the reconstituted human immune system by the cord blood donors in the NOG mice. As each group contains hu-NOG mice from two or more distinct donors, the variability in responses probably more closely mimics the diverse repertoire of patients’ immune responses in clinical trials than immune responses in syngeneic tumor mouse models. Importantly, a syngeneic mouse model B16F10 in C57BL/6 mice was considered.^56^ However, because of the following limitations this was concluded to be irrelevant: (1) the tested vectors are human adenoviruses, which do not replicate in murine cells optimally, and (2) the tested vectors encode human specific transgenes, TIMP2 and PAD1, so their efficacy could not be tested in syngeneic mouse model settings.

In summary, dual-expressing oncolytic adenoviruses had significantly higher anti-tumor activity than single-transgene oncolytic viruses and vehicle control. Treatment with each of the viruses significantly increased the percentage of activated cytotoxic T cells, and reactivation of T cells with anti-tumor activity during treatment of melanomas with immune checkpoint inhibitors is associated with improved outcomes among responders.^57^ These results support the further development of ONCOS-212 for cancer treatment of tumors sensitive to arginine deprivation, inhibition of angiogenesis, and disruption of tumor blood cell endothelial cell proliferation by modulation of MMPs.

## Materials and methods

### Viruses and reagents

Modifications of the investigated viruses are summarized in Table S1. The human TIMP2 gene (GenBank, #NM_003254.2) and the gene encoding the *Mycoplasma arginini* PADI1 (GenBank, #AB033768.2) protein^58^^,^^59^ obtained from Origene Technologies and Integrated DNA Technologies were inserted into our Ad5/3 backbone to generate the indicated viruses (Table S1). ONCOS-207 (TIMP2), ONCOS-209 (PADI1), ONCOS-207+ONCOS-209, ONCOS-210 (PADI1-P2A-TIMP2), and ONCOS-212 were grown on 293 and/or A549 cells. The virus was constructed, characterized by OD260 Inc (Boise, ID). The concentration of total viral particles was assessed by measurements with UV/Vis spectrophotometry at 260 and 280 nm. VP was calculated using the formula OD260 reading × dilution factor × (1.1 × 10^12^ particles) = number of particles per milliliter of sample.

### Melanoma cells and reagents

The four human melanoma cell lines were purchased from the American Type Culture Collection (ATCC): A2058 (CRL-1147), A375 (CRL-1619), SK-MEL-2 (HTB-68), and SK-MEL-28 (HTB-72). Cells were grown in DMEM supplemented with 10% FBS and 1% penicillin/streptomycin. The two cell lines A375 and SK-MEL-2 were seeded in 24-well plates at 2.5 × 10^4^ cells/well, while A2058 and SK-MEL-28 were seeded at 1.5 × 10^4^ cells/well for the following assays: flow cytometry for annexin IV, calreticulin exposure, ATP release, and HMGB1 release. Cells received the indicated treatment on the day of plating.

### Cell viability

Cells (5,000 cells/well) were seeded in 96-well plates in 200 μL medium and treated as indicated on the same day. Viability was assessed 72 h after treatment with the MTS assay kit (catalog number ab197010; Abcam), according to the manufacturer’s instructions.

### ELISA analyses

A549 infected cells were harvested 96 h after infection with viruses (ONCOS-207, ONCOS-209, ONCOS-210, and ONCOS-212). Proteins were extracted from the frozen cell pellet using NP40 cell lysis buffer per the manufacturer’s guidelines (catalog number FNN0021; Invitrogen, Carlsbad, CA). Human TIMP2 (catalog number ELH-TIMP2-1; RayBiotech) and human PADI1 (catalog number E4080hu; BT-labs) concentrations were determined using ELISA according to manufacturer’s instructions using protein extracts.

### Analysis of apoptotic and necrotic cells

Cells were seeded in 24-well plate at a density of 2.5 × 10^4^ cells per well for A375 and SK-MEL-2 cell lines and at a density of 1.5 × 10^4^ cells per well for A2058 and SK-MEL-28 cell lines. Treatment was initiated on the same day of plating and the amount of apoptotic and necrotic cells was measured 72 h after the beginning of the treatment by flow cytometry, using the dead cell apoptosis kit (catalog number 10267392; Thermo Fisher Scientific). The kit consists of FITC, annexin V, and the red fluorescent propidium iodide (PI) nucleic acid binding dye. PI stains dead cells with red fluorescence, binding tightly to the nucleic acids in the cell. After staining a cell population with FITC, annexin V, and PI, apoptotic cells show green fluorescence, dead cells show red and green fluorescence, while live cells show little or no fluorescence. These populations were detected using a flow cytometer with FITC and PI channels.

### Flow cytometry

The effects of the four viruses on the apoptosis, calreticulin exposure, ATP release, and HMGB1 release were assessed in four melanoma cell lines. Briefly, A375 cells (2.5 × 10^4^/well), SK-MEL-2 (2.5 × 10^4^/well), A2058 (1.5 × 10^4^/well), and SK-MEL-28 (1.5 × 10^4^/well) were plated into flat-bottom 6-well plates and treated on the same day with the indicated viral inoculum. The number and percentage of cells that stained positive for the indicated marker was performed at 72 h post-treatment.

To measure calreticulin exposure, cells were stained for cell surface expression of calreticulin with anti-calreticulin antibody for 30 min at 4°C. Cells were washed and resuspended in flow cytometry buffer. Samples were assessed using the Attune Net flow cytometer.

The amount of apoptotic and necrotic cells was measured after 72 h by using the dead cell apoptosis kit according to the manufacturer’s instructions and the Attune Net flow cytometer.

### Assays for ATP release and HMGB1

The amount of ATP and HMGB1 released into the supernatant by 72 h was evaluated by using the ATP assay kit (ab83355; Abcam) and by ELISA (ST51011; International) according to the manufacturer’s instructions.

### A2058 tumor model in nude BALB/c mice

After approval of the animal protocol by the local animal care board, A2018 tumor cells (2 × 10^6^ cells, 100 μL in PBS) were injected in each flank of eight-week-old female BALB/c mice (Charles River). Tumor volumes in each flank were measured beginning on day −3 (12 days after tumor engraftment). Tumor volumes on day 14 after tumor inoculation informed the randomization of the 48 mice into six groups of eight mice (16 tumors/group) for tumor treatment the next day. Each tumor was injected with 50 μL PBS (vehicle) or 2.5 × 10^6^ VP/tumor of indicated virus that had been freshly prepared in PBS from frozen stock on days 1, 3, 5, 12, 19, and 26 post-randomization into groups. Mice were monitored daily for unexpected signs of distress (Table S3).

Body weight and tumor volume were assessed 3 times per week until sacrifice. Tumor dimensions were measured using a digital caliper and tumor volumes (square millimeters) were calculated according to the following formula: volume = π/6 × width^2^ x length. Mice were sacrificed if body weight declined by 20%, a tumor volume exceeded 1,000 mm^3^, or an animal health score of 6 was reached (Table S3).

### A2058 tumor model in humanized NOG mice

Three hypotheses were tested in the A2058 melanoma model in the humanized NOG mice: (1) the oncolytic viruses expressing the two transgenes would be more effective than an oncolytic expressing one transgene, (2) ONCOS-212 treatment would reduce growth of challenge of A2058 melanoma tumor, and (3) ONCOS-212 efficacy can be increased with a 200-fold higher dose.

Humanization was performed as previously described,^33^ and the humanization rates at randomization ranged from 30% to 82%. The median humanization rate as a mean for animal groups was between 54% and 55%. The mice were randomized into groups, and most groups were injected with 2 × 10^6^ A2058 tumor cells. The dose and treatment schedules for the groups are shown in Table S4. Briefly, the A2058 melanoma tumor cells (2 × 10^6^) were injected into both flanks on day −14. On the basis of the humanization rates and tumor volumes on day −1, mice were randomized into groups on day 0. Both tumors in all groups were injected with PBS (vehicle) or the indicated oncolytic virus and dose (2.5 × 10^6^ VP/tumor or high dose 5 × 10^8^ VP/tumor) on 1, 3, 5, 12, 19, and 26. Blood samples were obtained on day −1 and at the end of the study (Figure S3).

The abscopal effect of ONCOS-212 was investigated in the A2058 xenogeneic tumor model in humanized NOG and involved a challenge with A2058 (Figure S3B). Briefly, A2058 tumor cells (5 × 10^5^) were inoculated into the right flank only on day −14. ONCOS-212 (2.5 × 10^6^ VP; 50 μL) treatment began on day 1 in right flank tumors and proceeded as indicated (Figure S3B). On day 18, A2058 tumor cells (5 × 10^5^) were inoculated into the left flanks and their growth monitored for 24 days (to day 42). Growth of right and left tumors was compared.

### Biostatistics

GraphPad Prism software (version 7) was used to analyze all *in vitro* and *in vivo* variables. Statistical analysis included a repeated-measures t test and one- or two-way ANOVA with Tukey’s multiple comparison’s test.

The FTV calculation method was used to assess therapeutic synergism.^60^^,^^61^ Briefly, the observed FTV equaled the mean tumor volume for each experimental group divided by the mean tumor volume of the vehicle control group. The expected FTV for a combination (e.g., ONCOS-207 + ONCOS-209) equals the product of the observed FTV for the individual groups (expected FTV_ONCOS-207+ONCOS-209_ = FTV_ONCOS-207_ × FTV_ONCOS-209_). The ratio of the expected FTV divided by the observed FTV indicated the nature of the interaction: >1 indicated synergism, 1 indicated additive, and <1 indicated less than additive (antagonism). Spearman correlation coefficient was used to identify potential relationships between individual tumor volume and percentage of CD8+ T cells in the tumor-infiltrating lymphocytes per gram of tumor tissue.

## Data Availability

Data are available on request from the corresponding author (L.K.).

## Supplemental information

Document S1. Figures S1–S6 and Tables S1–S4

Document S2. Article plus supplemental information
