# Supplementary material for: Next generation oncolytic viruses expressing PADI1 and TIMP2 exhibit anti-tumor activity against melanoma in nude and humanized mouse models
Source: Mol Ther Oncolytics. 2023 Jan 13;28:158–70. doi: 10.1016/j.omto.2023.01.002 (PMC9922816; doi:10.1016/j.omto.2023.01.002)
Supplement: Document S1. Figures S1–S6 and Tables S1–S4 [file mmc1.pdf]

OMTO, Volume *28*

Supplemental information

Next generation oncolytic viruses expressing PADI1 and TIMP2 exhibit anti-tumor activity against melanoma in nude and humanized mouse models

Lukasz Kuryk and Anne-Sophie W. Møller

Table S1. Summary of the genetic modifications in viral structure in the oncolytic viruses.

Name	E1	E3	Fiber
ONCOS-207	Deleted 24 bp	TIMP2 (ΔBsiWI-Mfel)	Ad5/3
ONCOS-209	Deleted 24 bp	PADI1 ((ΔBsiWI-Mfel)	Ad5/3
ONCOS-210	Deleted 24 bp	PADI1-P2A-TIMP2 (ΔBsiWI-Mfel)	Ad5/3
ONCOS-212	Deleted 24 bp	CMV-PADI1-IRES-TIMP2-SV40pA (ΔBglII)	Ad5/3

Table S2. Assessment of therapeutic synergism in the A2058 model in nude BALB/c mice with the fractional tumor volume (FTV) calculation method.

Tumor growth (days)	Days post Tx[a] initiation	FTV		ONCOS-210			ONCOS-212			ONCOS-207 + ONCOS-209		
		ONCOS-207	ONCOS-209	Exp[b] FTV	Obs[c] FTV	Ratio Exp/Obs	Exp[b] FTV	Obs FTV	Ratio Exp[b]/Obs	Exp FTV	Obs FTV	Ratio Exp[b]/Obs
35	21	1.35	1.48	2.00	1.01	**1.97**	2.00	0.92	**2.16**	2.00	0.86	**2.32**
47	33	0.89	0.99	0.87	0.56	**1.55**	0.87	0.55	**1.59**	0.87	0.64	**1.35**

[a]Tx, treatment; [b]Exp, expected; [c]Obs, observed

Table S3. Characteristics of clinical signs in animal health scoring.

Clinical sign	Abb.	Description	Score
Coat	C	Normal	0
		Lack of grooming, partial alopecia	1
		Massive alopecia, wounds, bleedings, inflammation	2
Movement	M	Normal	0
		Slow movement, paralysis of one member	2
		Difficulties to eat or drink, paralysis of more than one member	3
Activity	A	Normal	0
		Agitated, over-reactive, hypo-reactive	1
		Prostrated	3
Paleness	P	Normal	0
		Slight (no Ear blood vessels visible)	1
		Severe (Ear + Feet)	2
Body weight	W	Normal	0
		Segmentation of the vertebral column evident, pelvic bones palpable	2
		Emaciated, skeletal structure prominent	3

Table S4. Treatment characteristics in A2058 tumor models in humanized NOG mice.

#	Name	N	A2058 injection, $2x10^6$/flank	Dose in Left flank tumor	Dose in right flank tumor	Schedule (days)
**Comparison of efficacy of oncolytic viruses expressing PAD1, TIMP2, or both genes**						
1	Vehicle (PBS)	8	Day -14	PBS (50 µL)	PBS (50 µL)	1, 3, 5, 12, 19, 26
2	ONCOS-210	8	Day -14	$2.5x10^6$ VP	$2.5x10^6$ VP	1, 3, 5, 12, 19, 26
3	ONCOS-212	8	Day -14	$2.5x10^6$ VP	$2.5x10^6$ VP	1, 3, 5, 12, 19, 26
4	ONCOS-207	8	Day -14	$2.5x10^6$ VP	$2.5x10^6$ VP	1, 3, 5, 12, 19, 26
5	ONCOS-209	8	Day -14	$2.5x10^6$ VP	$2.5x10^6$ VP	1, 3, 5, 12, 19, 26

6	ONCOS-207 + ONCOS-209	8	Day -14	$2.5x10^6$ VP	$2.5x10^6$ VP	1, 3, 5, 12, 19, 26
**Abscopal effect of ONCOS-212**						
7	ONCOS-212	8	Right flank, Day -14, $5x10^5$ cells; Left flank, Day +18, $5x10^5$ cells	-	$2.5x10^6$ VP	1, 3, 5, 12, 19, 26
**Effect of 300-fold higher dose of ONCOS-212**						
8	ONCOS-210	6	Day -14	$5x10^8$ VP	$5x10^8$ VP	1, 3, 5, 12, 19, 26
9	ONCOS-212	6	Day -14	$5x10^8$ VP	$5x10^8$ VP	1, 3, 5, 12, 19, 26

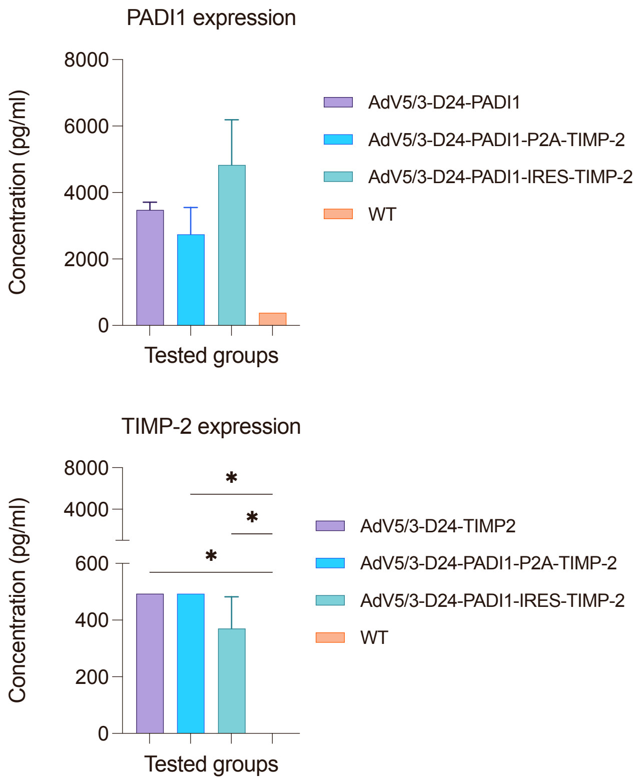

Figure S1. Expression level of PAD1 and TIMP-2 transgenes by ONCOS-207, ONCOS-209, ONCOS-210, and ONCOS-212 treated cells. The expression of TIMP-2 and PADI1 from ONCOS-207, ONCOS-209, ONCOS-210 and ONCOS-212 was assessed by infecting A549 cells with the virus, harvesting proteins 96 hours after the infection and detecting TIMP-2 and PADI1 by ELISA. Protein extracts concentration was determined by Elisa according to the manufacturer's instructions. Results are expressed as mean ± SEM. One-Way ANOVA was used to compare the groups with Tukey's multiple comparison's test. * $P < 0.05$.

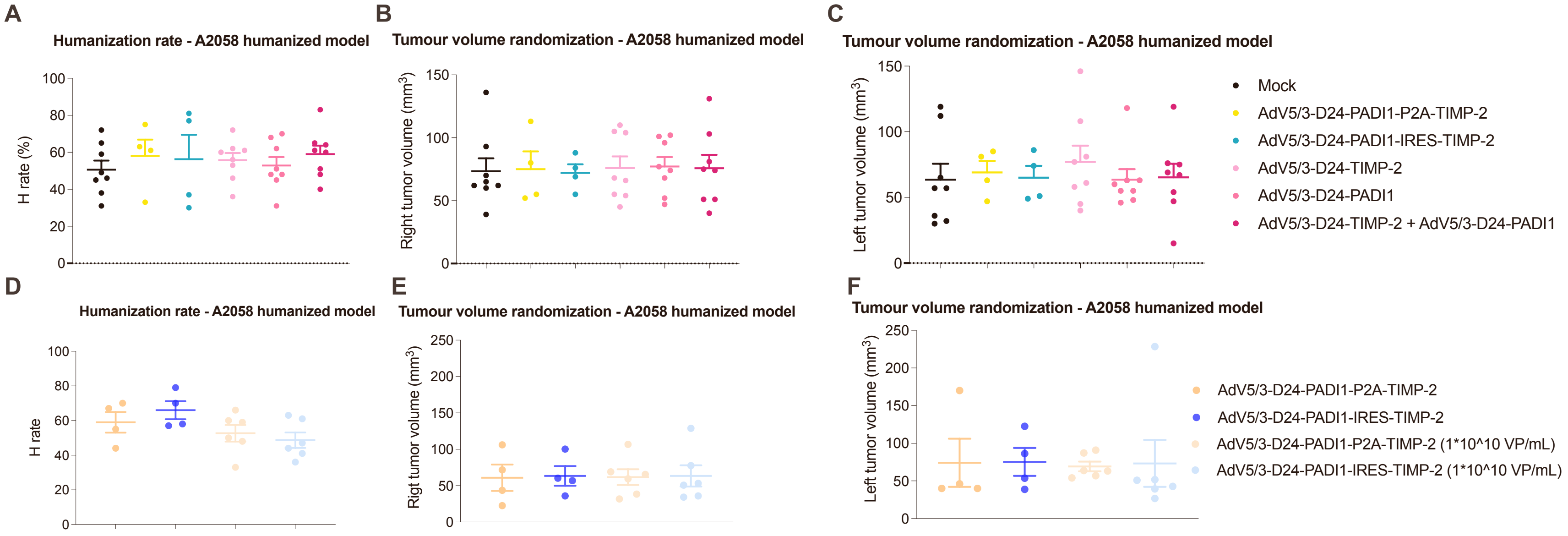

Figure S2. Randomization of the groups in the A2058 tumor model in humanized NOG mice were based on both tumor volumes and humanization rates present in the individual mice. One-way ANOVA with Tukey's multiple comparison's test was used.

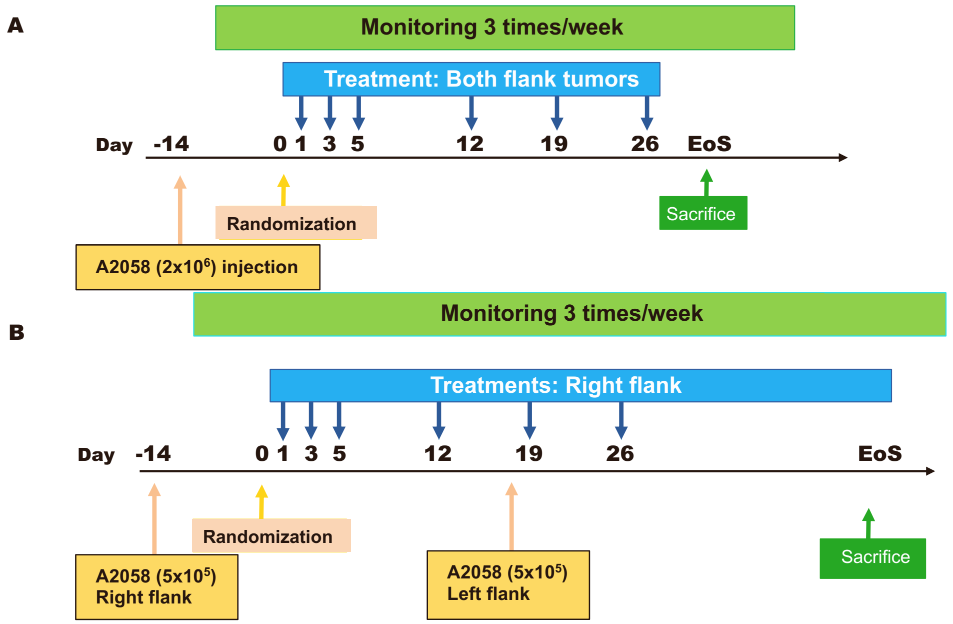

Figure S3. Summary of the schedules for tumor implantation, randomization, blood sampling, oncolytic viral treatments, and monitoring for tumor volumes and clinical health scores in the A2058 tumor model in the humanized NOG mice.

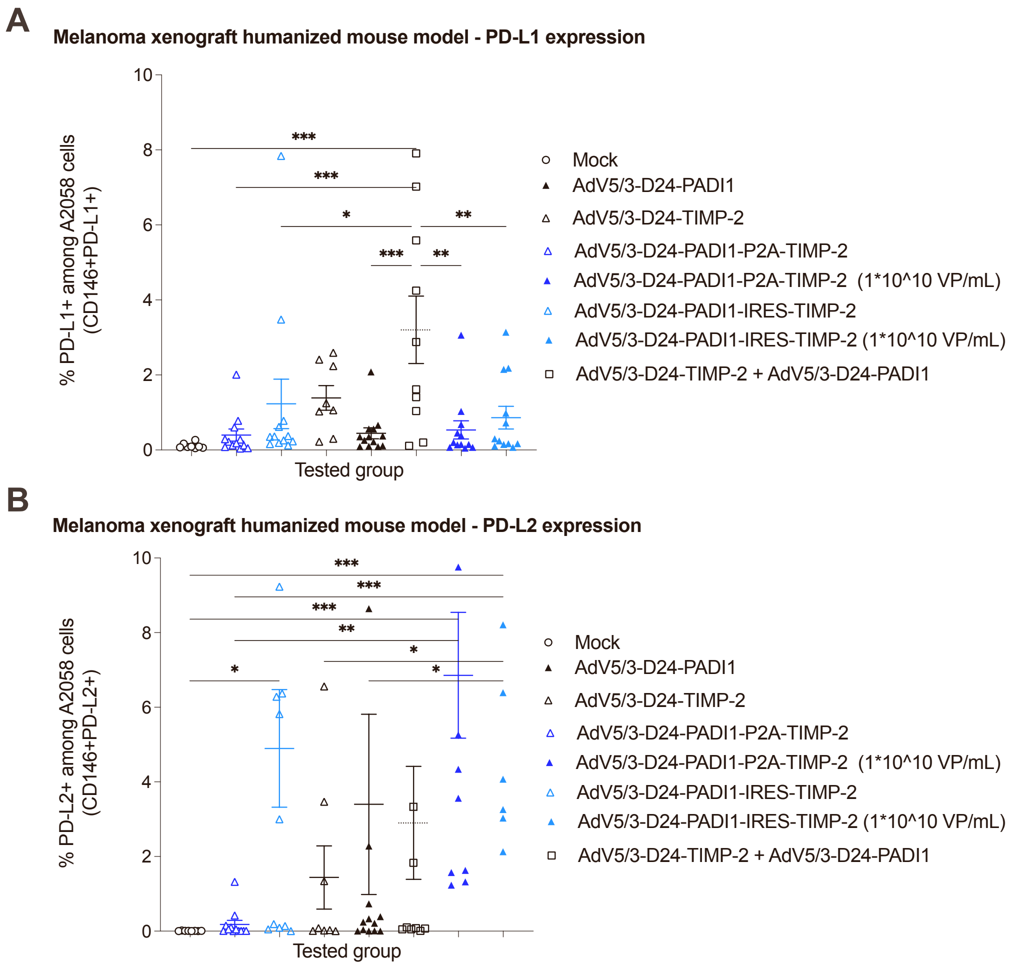

Figure S4. Oncolytic viral treatment increased the percentage of PD-L1 and PD-L2 positive A2058 tumor cells. A2058 population was defined as CD146+. A. Percentage of A2058 cells expressing PD-L1. B. Percentage of A2058 cells expressing PD-L2. Individual data and mean ± SEM are presented for each group. One-way ANOVA with Tukey's multiple comparison's test was used. * $P \leq 0.05$; ** $P \leq 0.01$; *** $P \leq 0.001$.

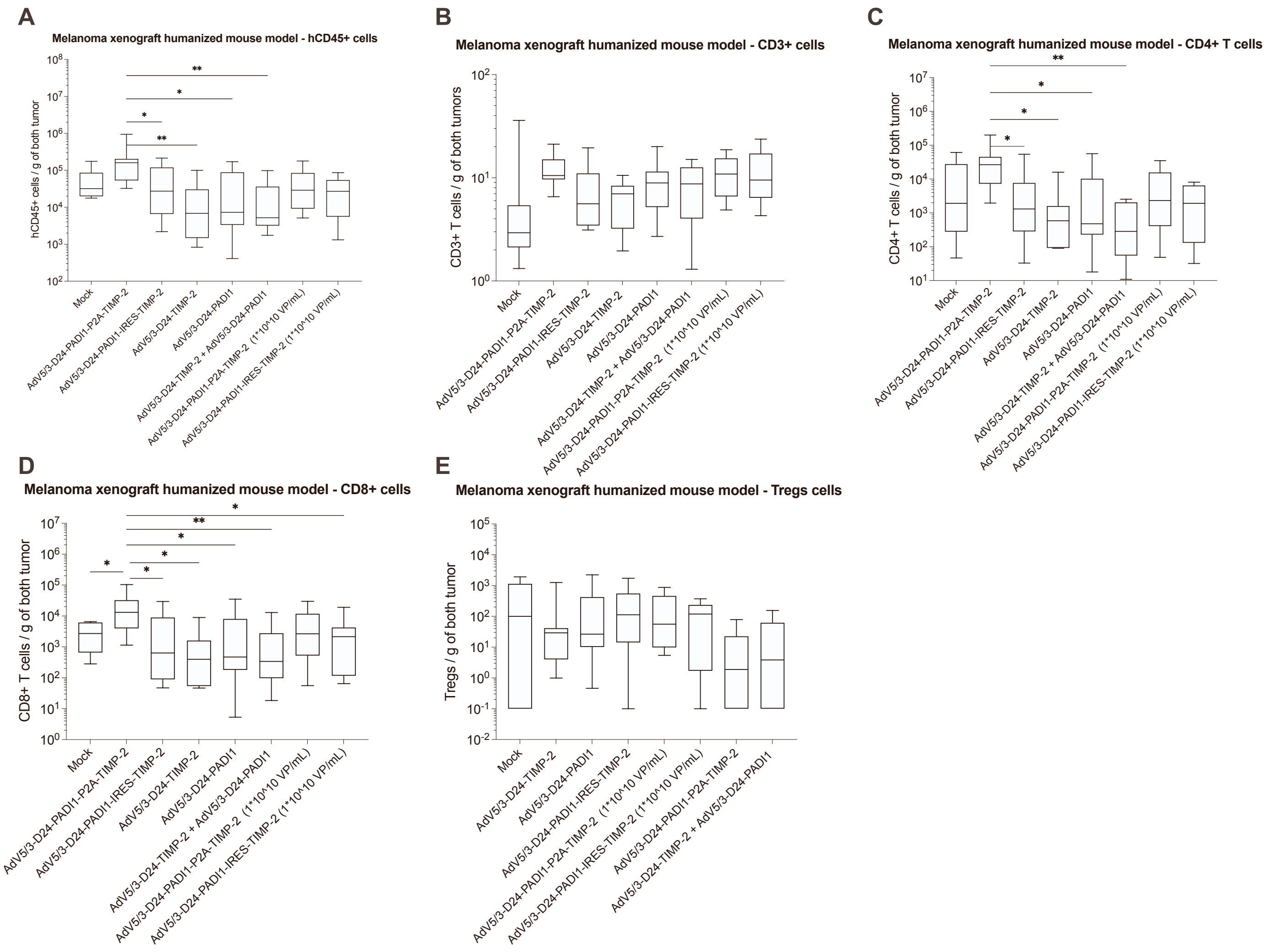

Figure S5. Immune infiltration phenotype in the tumors at sacrifice. A: Human CD45 leukocytes. B: T cells. C: CD4+ T cells. D: CD8+ T cells. E: Treg cells. Immune infiltration was analyzed by flow cytometry in the tumors at sacrifice. N = 6-12 tumors per group. Individual data are presented as box with wishers (min to max). One-way ANOVA with Tukey's multiple comparison's test was used. * P ≤ 0.05; ** P ≤ 0.01.

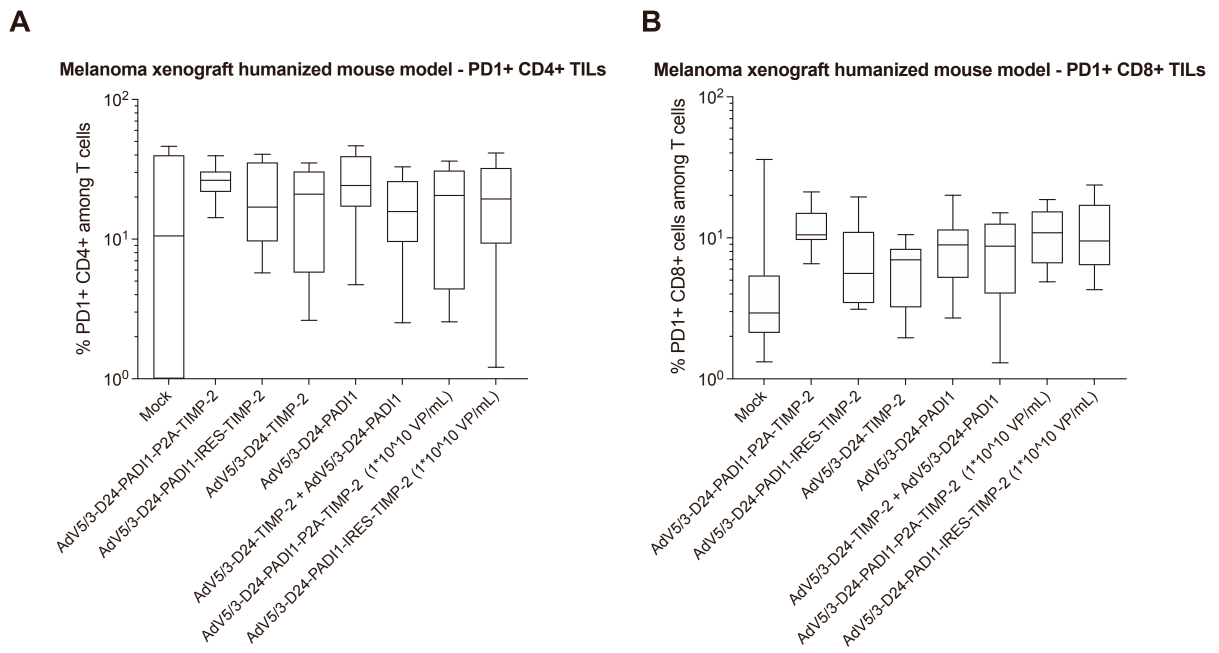

Figure S6. PD1 expression in infiltrated CD4+ and CD8+ T cells populations. Data from right and left tumor were combined. A. percentage of infiltrated CD4+ T cells expressing PD1 among total CD3+ T cells. B, percentage of infiltrated CD8+ T cells expressing PD1 among total T cells. N = 6-12 tumors per group. Individual data are presented as box with wishers (min to max). One-way ANOVA with Tukey's multiple comparison's test was used. * $P \leq 0.05$.
